# Mechanisms underlying persistent liver fibrosis progression in *Schistosoma*-infected individuals post-treatment

**DOI:** 10.1186/s40249-025-01373-x

**Published:** 2025-10-14

**Authors:** Zhigang Lei, Sha Zhou, Chuan Su, Guanling Wu

**Affiliations:** 1 Department of Pathogen Biology, Nanjing Medical University, Nanjing, 211166 China; 2 Key Laboratory for Pathogen Infection and Control of Jiangsu Province, Nanjing, 211166 China

**Keywords:** Schistosomiasis, Liver fibrosis progression, Post-treatment, Praziquantel

## Abstract

**Background:**

Schistosomiasis, an infectious disease of poverty, remains a public health challenge worldwide. Although praziquantel chemotherapy has been proven to be an effective antiparasitic intervention, real-world evidence indicates that in patients with hepatointestinal schistosomiasis, hepatic fibrosis may continue to progress even after treatment. The current understanding of the mechanisms underlying persistent liver fibrosis progression in *Schistosoma*-infected individuals post-treatment is unclear. The aim of this commentary is to analyze the critical yet multifactorial determinants contributing to the persistent progression of liver fibrosis and to advocate for a comprehensive research focus to support the global elimination of this disease.

**Main text:**

Multiple mechanisms may contribute to the persistent progression of liver fibrosis in schistosomiasis. These include the continued presence of viable *Schistosoma* eggs, co-infection with hepatitis viruses, alterations in splenic structure and function, disruption of the intestinal mucosal barrier, hepatic ischemia and hypoxia, hepatocyte death, specific types of collagen deposition, and host genetic variations. However, additional factors potentially contributing to host pathology warrant further investigation.

**Conclusions:**

In the post-schistosomiasis control era, expanding the focus of research to include the “post-treatment” phase is essential. Investigating the mechanisms underlying the persistent progression of liver fibrosis and identifying future research priorities may enhance efforts toward the global elimination of schistosomiasis and improve long-term health outcomes for individuals who have received praziquantel treatment.

**Graphical Abstract:**

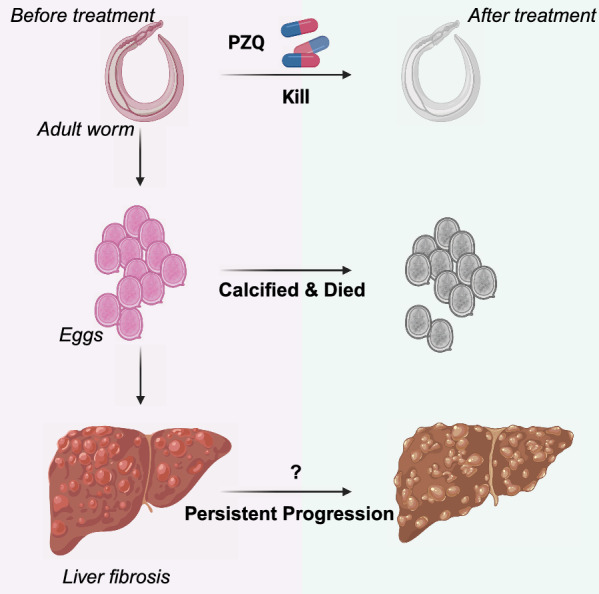

## Background

The World Health Organization (WHO) has set an ambitious goal to eliminate schistosomiasis, a neglected tropical disease posing urgent public health challenges, worldwide by 2030 [1]. Schistosomiasis is a snail-borne infectious disease caused by trematode blood flukes of the genus *Schistosoma*. Among the species within this genus, *S. japonicum*, *S. mekongi* and *S. mansoni*, which are predominantly distributed in Southeast Asia, Africa, and South America, are the main causative agents of hepatointestinal schistosomiasis. The deposition of *Schistosoma* eggs in the presinusoidal periportal spaces of the liver induces granulomatous inflammation, leading to hepatic fibrosis, hepatomegaly, portal hypertension, hypersplenism, and in severe cases, death [2]. An uncontrolled granulomatous immune response can drive disease progression to an advanced stage known as chronic schistosomiasis. Researchers in China refer to this specific stage as “advanced schistosomiasis”, which is clinically characterized by distinct phenotypes, including the massive splenomegaly type, ascitic type, colonic proliferative type, and/or dwarfism type. Chronic/advanced schistosomiasis typically requires 5 to 10 years or even longer to develop and represents the most severe clinical manifestation of the disease [2–4]. In China, epidemiological data from the past two decades indicate that the number of newly reported indigenous cases of *S. japonicum* infection has steadily decreased, approaching zero. However, the number of advanced schistosomiasis cases has remained high, which may be related to the progression of a large number of praziquantel (PZQ)-treated infections to the advanced stage in the last century [5]. Notably, although the WHO’s global strategy for schistosomiasis elimination includes key components such as prevalence thresholds, snail control, and diagnostic improvements, it does not specifically target the unresolved issue of continued hepatic fibrosis progression or other long-term complications in patients following PZQ treatment. Therefore, given the goal to eliminate the schistosomiasis globally, schistosomiasis control efforts are facing a turning point and new challenges, with chronic/advanced schistosomiasis likely to be one of the most difficult issues in the “post-schistosomiasis control era”.

The soluble egg antigen (SEA) produced by miracidia within the *Schistosoma* eggs is phagocytosed and processed by hepatic macrophages and other antigen-presenting cells, which in turn activate and recruit immune cells—such as eosinophils, T lymphocytes, neutrophils, and macrophages—to the liver to encapsulate the eggs and form egg granulomas. These immune cells secrete large amounts of cytokines, chemokines, and damaging factors that can activate hepatic stellate cells (HSCs), leading to excessive collagen production and ultimately resulting in liver fibrosis [6, 7]. Thus, SEA serves as a pivotal trigger in the pathogenesis of hepatic fibrosis during schistosomiasis. Theoretically, following PZQ chemotherapy, adult worms are eliminated, and the remaining eggs in hepatic granulomas gradually die and calcify over the course of several months. As SEA is cleared from the host, the progression of liver fibrosis is expected to cease. However, numerous studies based on ultrasonography and/or serologic tests, in both human and animal models have demonstrated that PZQ treatment does not fully halt fibrosis progression in all infected individuals. In other words, even after the successful elimination of adult worms and death of viable eggs, hepatic fibrosis can continue to progress in some hosts over the long term, leading to severe clinical consequences [8–10]. Although a more modern diagnostic and analytical technique is highly recommended for liver fibrosis investigations, the mechanisms underlying this persistent fibrosis remain poorly understood. Here, some relevant studies are summarized in the Table 1.

**Table 1:** Follow-up of liver fibrosis progression in *Schistosoma*-infected individuals post-treatment

Study (Author, Year)	Country/Region	*Schistosoma* species	Treatment (Drug/Dose)	Follow-up duration	Fibrosis assessment	Key findings
Li et al., 2002 (PMID: 12,497,984) [9]	China	*S. japonicum*	Praziquantel (40 mg/kg)	5 years	Ultrasound assessment, stool examination	Some patients developed fibrosis despite substantial reductions in egg counts
Peter M. Wiest et al., 1994 (PMID: 8,074,249) [11]	China	*S. japonicum*	Praziquantel	3 years	Ultrasonography	No change in the community prevalence of *Schistosoma*-induced hepatic fibrosis was observed
Peter M. Wiest et al., 1993 (PMID: 8,236,395) [12]	China	*S. japonicum*	Praziquantel	3 years	Ultrasonography	Persistence of hepatomegaly and *Schistosoma*-induced periportal fibrosis in adults
Tanaka M, 1992 (PMID: 1,343,908) [13]	Philippines	*S. japonicum*	Praziquantel (3 × 20 mg/kg)	Variable	Ultrasonographical examination, biochemical serum analysis	The significant ultrasonographical changes could not be detected in the patients with severe hepatic fibrosis caused in the long-term infection
Elizabeth J. Carlton et al., 2010 (PMID: 20,502,515) [14]	China	*S. japonicum*	Praziquantel	Up to five years	Ultrasound examination	Only 28% of individuals with severe parenchymal fibrosis (grades 2 or 3) at enrollment reversed to normal or grade 1 within five years
Siddig A Rahoud et al., 2010 (PMID: 20,030,714) [15]	Sudan	*S. mansoni*	Praziquantel	39 months	Ultrasound evaluation	Periportal fibrosis progressed to higher grades (13.6%), or did not change in 50.8% patients
Nega Berhe et al., 2008 (PMID: 18,256,420) [16]	Ethiopia	*S. mansoni*	Praziquantel (40 mg/kg)	26 months	Ultrasonography, serologic tests	Subjects with severe periportal thickening/fibrosis showed no improvement
K Frenzel et al., 1999 (PMID: 10,403,322) [17]	Uganda	*S. mansoni*	Praziquantel (40 mg/kg)	2.7 years	Ultrasound	No significant difference was detected between repeated praziquantel treatment and single dose praziquantel treatment with respect to reversibility of periportal thickening
Raiza Ruiz-Guevara et al., 2007 (PMID: 17,992,403) [18]	Venezuela	*S. mansoni*	Praziquantel (40 mg/kg)	3 or 5 years	Parasitological, serological, clinical and ultrasoundevaluations	Patients with more advanced schistosomiasis did not present a reversal in periportal fibrosis
E I Odongo-Aginya et al., 2010 (PMID: 23,878,699) [19]	Uganda	*S. mansoni*	Praziquantel (40 mg/kg)	1 year	Ultrasonography	All stage of periportal fibrosis were evident after treatment
H Keang et al., 2007 (PMID: 17,568,642) [20]	Cambodia	*S. mekongi*	Praziquantel (40 mg/kg)	7 years	Ultrasonography	Periportal fibrosis with portal hypertension was diagnosed in 46% of people treated with praziquantel in this area
Hu et al., 2021 (PMID: 34,959,487) [10]	China	*S. japonicum*	Praziquantel (40 mg/kg)	24 years	Ultrasonography	Among 292 residents examined both in 1995 and 2019, liver damage was stable in 141 (48.29%), whereas 86 (29.45%) developed severe liver fibrosis
Olveda et al.,2017 (PMID: 27,816,660) [21]	Philippines	*S. japonicum*	Praziquantel (40 mg/kg)	2 years	Ultrasonography	Among 424 patients with all stages of fibrosis (excluding the advanced stage grade III), a quarter of individuals presented progressive liver parenchyma grading after praziquantel treatment

Therefore, investigating the causes and mechanisms responsible for the chronic and progressive liver fibrosis observed in schistosomiasis patients after PZQ treatment is critically important. Such research is essential for consolidating the gains made in schistosomiasis control, improving patient outcomes, and supporting long-term public health and socioeconomic development. In this commentary, several potential mechanisms contributing to the continued progression of liver fibrosis following PZQ therapy are proposed, and perspectives and recommendations are offered for intervention strategies to mitigate schistosomiasis-related hepatic fibrosis.

### Mechanistic overview of the post-treatment progression of schistosomiasis-associated hepatic fibrosis

Multiple factors, either individually or synergistically, can exacerbate the progression of hepatic fibrosis in schistosomiasis patients after treatment. In Table 2, we summarize the mechanisms through which different factors may induce or exacerbate hepatic fibrosis, and propose potential intervention strategies.

**Table 2:** Potential mechanisms underlying persistent liver fibrosis progression in schistosomiasis patients after praziquantel treatment

Mechanism category	Potential factors/mechanisms	Pathophysiological consequences	Potential interventions
Persistent presence of viable eggs	∙Incomplete clearance of adult worms ∙Re-infection ∙Praziquantel resistance	Continuous release of SEA → sustained immune activation → HSC activation and collagen deposition	∙Repeated/periodic PZQ administration ∙Improved diagnostic and monitoring tools
Co-infection with hepatitis viruses	∙HBV and/or HCV	Suppressed Th1 immunity, enhanced viral replication → exacerbated inflammation and fibrosis	∙Antiviral therapy (e.g., entecavir) ∙Prevention of blood-borne transmission
Alterations of splenic structure and function	∙Splenomegaly ∙Hypersplenism ∙Lymphoid follicle remodeling	Cytopenia, impaired immunity, altered cytokine responses → worsened liver injury and fibrosis	∙Splenectomy in selected patients ∙Modulation of immune and platelet function
Disruption of intestinal mucosal barrier	∙Dysbiosis ∙Epithelial barrier dysfunction	Microbial translocation (e.g., LPS, bile acids) → activation of TLR4–MyD88–NF-κB/TGF-β pathways → HSC-driven fibrogenesis	∙Gut microbiota modulation (probiotics, FMT) ∙Reduction of endotoxin burden
Hepatic ischemia, hypoxia, and hepatocyte death	∙Vascular obstruction ∙ROS overproduction ∙Hepatocyte death ∙HMGB1 release	ROS and DAMPs trigger HSC activation and immune-mediated fibrogenesis	∙Antioxidants (e.g., apocynin) ∙HMGB1 inhibitors (glycyrrhizin, diclofenac)
Collagen deposition subtype shift	∙Transition from type III (more degradable) to type I collagen (less degradable)	Reduced reversibility of fibrosis, progression to advanced stage	∙Early antiparasitic therapy ∙Monitoring of collagen subtypes
Host genetic susceptibility	∙HLA alleles ∙ABO blood system ∙T cell receptor repertoire	Individual variation in fibrosis severity independent of infection load	∙Genetic screening ∙Personalized management strategies

*SEA* Soluble egg antigen, *PZQ* Praziquantel, *HSC* Hepatic stellate cell, *HBV* Hepatitis B virus, *HCV* Hepatitis C virus, *Th1* T helper type 1, *LPS* Lipopolysaccharide*, TLR4* Toll-like receptor 4, *MyD88* Myeloid differentiation primary response 88, *NF-κB* Nuclear factor kappa-B, *TGF-β* Transforming growth factor beta, *FMT* Fecal bacteriotherapy, *ROS* Reactive oxygen species, *DAMPs* Damage-ssociated molecular patterns, *HMGB1* High mobility group box 1, *HLA* Human leukocyte antigen

### Persistent presence of viable eggs

In the host, adult *Schistosoma* worms typically have a lifespan of 3 to 10 years, with rare cases of survival lasting up to 40 years [22–24]. PZQ therapy, which targets adult worms residing in blood vessels, is a fundamental component of schistosomiasis treatment, achieving cure rates between 70 and 100% following a single treatment course [25]. However, in endemic areas with high prevalence, the risk of reinfection is substantial, particularly among individuals in occupations involving frequent contact with infested water sources [9, 26]. Moreover, PZQ is highly effective against adult worms but has little to no effect on *Schistosoma* eggs and has only limited efficacy against immature worms [27]. Therefore, if treatment is administered too early, or if infection occurs at different times or larvae develop asynchronously, a single round of chemotherapy may result in suboptimal worm clearance. In such endemic regions, multiple or periodic treatments are recommended.

Although annual PZQ treatment is recommended for individuals residing in areas where schistosomiasis prevalence is high, concerns about potential drug resistance have emerged because of the reduced response to PZQ that has been reported in some areas during the past years [28, 29]. The possibility that the drug may have failed to completely eliminate all *Schistosoma* from the infected individual cannot be ruled out. On the other hand, in regions where intestinal schistosomiasis is endemic, the WHO recommends using the Kato-Katz technique [1]—which involves microscopic examination to calculate the number of eggs per gram of stool—to assess the prevalence and intensity of infection with *S. mansoni*, *S. mekongi* and *S. japonicum*. Egg detection is cost-effective and relatively simple to perform; however, it has several limitations, such as low sensitivity—especially in cases of mild infection—as well as intra- and inter-sample variability caused by the uneven distribution of eggs and fluctuations in egg excretion across different days [30]. Implementing a multifaceted screening approach, such as combining imaging, immunological, and artificial intelligence-based methods, may help increase the detection rate of false-negative schistosomiasis cases in high-risk populations.

### Co-infection with hepatitis virus

In countries or regions where schistosomiasis is endemic, co-infection with hepatitis B virus (HBV) and/or hepatitis C virus (HCV) is common among schistosomiasis patients. Epidemiological data from Egypt have shown that 63.8% and 27.3% of schistosomiasis patients are co-infected with HBV and HCV, respectively [31, 32]. In Brazil, the rates of co-infection with HCV and HBV among schistosomiasis patients are 12.9% and 15.8%, respectively [33]. Studies from China have also reported that approximately half of schistosomiasis patients are co-infected with HBV, whereas the co-infection rate with HCV is only 5.88% [34, 35]. Compared with healthy blood donors or even those with mild schistosomiasis from the same geographic region, patients with chronic or advanced hepatosplenic schistosomiasis are 7 to 10 times more likely to be co-infected with HBV [36]. Blood transfusions, surgery, endoscopic procedures, and parenteral nutrition are considered major risk factors for hepatitis virus infection in schistosomiasis patients, especially in those with advanced disease [37–39]. For example, in Egypt, a massive outbreak of HCV infection occurred among schistosomiasis patients during anti-schistosomiasis “mass campaigns”, primarily because of the repeated use of inadequately sterilized syringes [40].

Studies have shown that in patients co-infected with HBV and/or HCV and schistosomiasis, cell-mediated immune responses are significantly suppressed [41]. In chronic and advanced schistosomiasis, the immune response in the liver is predominantly Th2-mediated, whereas Th1 responses are inhibited, leading to accelerated replication of hepatitis viruses [42, 43]. Compared with patients with HBV infection alone, those co-infected with chronic schistosomiasis and HBV more often present persistent viremia, higher levels of transaminases and bilirubin, and more severe clinical manifestations of liver fibrosis or cirrhosis, such as ascites, jaundice, palmar erythema, and gynecomastia [36]. Clinical studies have also shown that incorporating antiviral treatment with entecavir into a regimen for chronic/advanced schistosomiasis patients co-infected with HBV can significantly improve liver cell injury and reduce hepatic fibrosis [44].

However, most existing studies are based on epidemiological observations, and although poorer prognoses have been noted in schistosomiasis patients co-infected with hepatitis viruses [33, 45], causal relationships are difficult to establish through human studies. Furthermore, owing to the lack of suitable animal models that replicate co-infection with *Schistosoma* and HBV or HCV, experimental investigations into the role and mechanisms of hepatitis virus infection in the progression of schistosomiasis-associated hepatic fibrosis remain limited.

### Alterations in splenic structure and function

Splenomegaly is one of the typical clinical manifestations in patients with chronic or advanced schistosomiasis. Approximately 30% of the blood entering the liver via the portal vein originates from the spleen. When hepatic fibrosis occurs in the late or uncompensated stage, intrahepatic portal blood flow is impeded, leading to congestive splenomegaly and subsequent cytopenias of varying degrees and types, a condition known as secondary hypersplenism. Owing to a prolonged disease duration and significant splenic enlargement, patients with chronic/advanced schistosomiasis often present with pancytopenia [46, 47]. Leukopenia increases susceptibility to infections, particularly hepatotropic viral infections. Additionally, reduced red blood cell counts compromise the regenerative capacity of hepatocytes, thereby exacerbating liver injury and fibrosis. Clinically, splenectomy is a common treatment strategy for managing massive splenomegaly in these patients [48]. Follow-up studies on patients undergoing splenectomy for chronic/advanced schistosomiasis have shown that, even without standardized anti-fibrotic therapy, liver fibrosis progressively improves as observed via ultrasound, and peripheral blood counts and liver biochemical parameters return to normal ranges [49].

Although clinical studies have demonstrated improvements in fibrosis markers such as type IV collagen and tissue inhibitor of matrix metalloproteinases (TIMP-1) following splenectomy [50], the mechanisms underlying the alleviation of fibrosis remain unclear. Animal experiments have shown that in mice with chemically induced hepatic fibrosis, splenectomy results in increased accumulation of Ly6C^low^ macrophages in the liver [51], which are known to secrete matrix metalloproteinases (MMPs) that help resolve fibrosis [52]. Additionally, the restoration of platelet levels in the liver after splenectomy [51] has been associated with the inhibition of transforming growth factor-β (TGF-β) gene transcription and increased MMP-9 expression, both of which contribute to the mitigation of hepatic fibrosis [53]. Although splenectomy has proven to be a cost-effective and efficient intervention for hepatic fibrosis in patients with chronic or advanced schistosomiasis, not all patients develop fibrosis regression or reversal after surgery [54], indicating the existence of other contributing factors that remain to be clarified.

In addition to splenomegaly and hypersplenism, *Schistosoma* infection can induce structural alterations in splenic lymphoid follicles, which play a critical role in the development and functional maturation of lymphocytes [55]. During the chronic phase of infection, splenic lymphocytes may undergo apoptosis, migrate into hepatic granulomas, or be phagocytosed by monocytes, leading to the gradual disappearance of lymphoid follicles and weakened immune function [56–58]. This immunosuppression may, to some extent, slow the progression of hepatic granuloma formation and fibrosis. However, studies have shown that when *Schistosoma* eggs are deposited in the splenic cortex and granulomas form, lymphoid follicles can be reconstructed by recruiting new lymphocytes or by competing with hepatic granulomas for lymphocyte chemotaxis [59]. Furthermore, splenic granulomas can stimulate the secretion of Th2-type cytokines, such as interleukin-4 (IL-4) and IL-5 [59], which further promote the progression of hepatic fibrosis.

### Disruption of the intestinal mucosal barrier

More than half of the eggs produced by adult *S. mansoni*, *S. mekongi* and *S. japonicum* are deposited in the intestinal wall, leading to not only hepatic damage but also to intestinal schistosomiasis. The symptoms of intestinal schistosomiasis are related primarily to the migration or entrapment of *Schistosoma* eggs in intestinal tissue. Granulomatous inflammation is commonly observed in the intestinal mucosa and is often accompanied by micro-ulcerations, superficial hemorrhages, and occasionally pseudopolyposis, resulting in damage to the intestinal mucosal barrier [26]. 16S rRNA sequencing analyses have revealed that, compared with healthy individuals, patients with advanced schistosomiasis and hepatic fibrosis present dysbiosis in both the composition and function of the gut microbiota [60]. This microbial imbalance further disrupts tight junctions between intestinal epithelial cells, suppresses mucin production, reduces the secretion of antimicrobial peptides, and promotes the overgrowth of pathogenic bacteria. These changes intensify intestinal inflammation and exacerbate barrier dysfunction [61]. The compromised intestinal barrier facilitates the translocation of gut microbiota and their metabolites—including lipopolysaccharide (LPS), bile acids, endogenous ethanol, and short-chain fatty acids—into the systemic circulation, which plays a critical role in promoting hepatic fibrogenesis [62]. For example, LPS entering the liver through portal circulation can bind to Toll-like receptors (such as TLR4) on hepatocytes and HSCs, activating the MyD88-NF-κB signaling pathway. This activation increases the expression of inflammatory cytokines, promotes TGF-β/Smad3 signaling, and stimulates extracellular matrix production by HSCs, thereby exacerbating liver fibrosis [61]. In addition, microbial antigens and other immunogenic substances from the gut can activate T-cell receptors (TCRs), modulate adaptive T-cell immune responses, and stimulate collagen synthesis in HSCs. Studies have shown that restoring gut microbiota homeostasis in *Schistosoma*-infected mice attenuates Th2-type immune responses in the liver and leads to a reduction in hepatic fibrosis [63].

### Hepatic ischemia & hypoxia and hepatocyte death

A large number of *Schistosoma* eggs are deposited in intrahepatic vessels, and the resulting granulomatous inflammation and fibrosis occur intravascularly. Varying degrees of vascular obstruction can lead to persistent hepatic ischemia and hypoxia in patients with chronic and advanced schistosomiasis. Under such conditions, hepatocellular mitochondria and the endoplasmic reticulum generate substantial amounts of reactive oxygen species (ROS), which are byproducts of incomplete oxygen reduction. ROS play essential roles in physiological signaling, gene regulation, host defense, and immune responses [64]. However, excessive ROS production induces oxidative stress in the liver and contributes to HSCs activation through multiple mechanisms. On the one hand, ROS can directly activate HSCs and promote their transdifferentiation into myofibroblasts, thereby increasing the synthesis of type I collagen and α-smooth muscle actin (α-SMA) [65]. On the other hand, ROS stimulate hepatocytes and HSCs to produce and activate TGF-β1 [66, 67], which in turn promotes fibrogenesis through both the canonical Smad2/4 pathway and non-Smad signaling cascades, such as the MAPK, ERK, p38, and JNK. These pathways induce connective tissue growth factor (CTGF) expression [68, 69] and increase the transcription of type I collagen genes (COL1A1 and COL1A2) [70], thus accelerating liver fibrosis progression. In addition, ROS can activate immune cells such as Kupffer cells, which secrete galectin-3 and other mediators that further promote HSCs activation [71]. In *Schistosoma*-infected mice, treatment with apocynin, an inhibitor of ROS production, significantly attenuated hepatic fibrosis [72].

To some extent, ischemia and hypoxia in the hepatic microenvironment can trigger various forms of hepatocyte death, such as pyroptosis, apoptosis, and necrosis [73–75]. Pyroptotic hepatocytes release inflammasome complexes such as NOD-like receptor thermal protein domain associated protein 3 (NLRP3) into the extracellular space, which can be engulfed by HSCs and induce their activation and collagen production [76]. Hepatocyte apoptosis, necrosis, and necroptosis have also been implicated in HSCs activation [77–79]. When necroptotic pathways involving receptor-interacting protein kinase 3 (RIPK3) and mixed lineage kinase domain-like protein (MLKL) are activated, hepatocytes release damage-associated molecular patterns (DAMPs) and ROS, which either directly activate HSCs or trigger hepatic immune cells to release inflammatory mediators, ultimately exacerbating liver fibrosis [80]. Nonetheless, the specific signaling pathways involved in the predominant forms of hepatocyte death during different stages of schistosomiasis, with or without living eggs, and potential therapeutic targets remain to be fully elucidated.

As a major DAMP, high mobility group protein B1 (HMGB1), which was originally identified as a chromatin-binding protein, has been shown to play critical roles in liver fibrosis progression. In various liver fibrosis-related diseases—for example, viral hepatitis, primary biliary cirrhosis, non-alcoholic steatohepatitis, drug-induced liver injury, and cholestatic liver disease—HMGB1 has been shown to translocate from the nucleus to the cytoplasm and eventually be secreted extracellularly [81]. Hypoxia is a major stimulus for HMGB1 expression and release [82–84]; however, the exact mechanisms through which hypoxia facilitates HMGB1 extracellular secretion remain unclear. In addition, hepatocytes are considered the primary source of HMGB1 in injured liver tissue [85]. Elevated levels of HMGB1 have been observed in the serum of patients with chronic schistosomiasis [86]. HMGB1 can bind to the receptor for advanced glycation end products (RAGE) on HSCs, which activates downstream signaling pathways, such as Smad2, PI3K/AKT, and ERK1/2, and thereby promotes HSCs proliferation, activation, and collagen production [81, 87]. Additionally, HMGB1 interacts with RAGE on group 2 innate lymphoid cells (ILC2), inducing the secretion of interleukin-13 (IL-13), which indirectly stimulates HSCs to produce more collagen and extracellular matrix proteins [88]. In *Schistosoma*-infected mice, the use of HMGB1 secretion inhibitors such as glycyrrhizin or diclofenac has been shown to reduce the production of type 2 cytokines (e.g., IL-13, IL-4 and IL-5), decrease the extent of hepatic fibrosis by approximately 50%, and improve survival [86]. Therefore, targeting HMGB1 expression and secretion may represent a promising strategy for controlling schistosomiasis-induced liver fibrosis.

### Other potential factors

During the progression of liver fibrosis, the synthesis of collagen fibers by HSCs occurs concurrently with the degradation of collagen by MMPs. However, compared with type III collagen, type I collagen is degraded at a significantly slower rate, indicating that the reversibility of liver fibrosis is closely associated with the specific type of collagen deposited [89]. In fact, the types of collagen fibers deposited in the liver vary across different stages of schistosomiasis infection. In the early stages, granulomas are primarily consist of type III collagen. As the infection progresses, the proportion of type I collagen increases and predominantly accumulates in the central regions of egg-induced granulomas to eventually become the dominant collagen type in the later stages of infection [90, 91]. Studies have shown that in advanced schistosomiasis, a lower type III to type I collagen ratio is associated with a reduced likelihood of fibrosis reversal [92]. Therefore, in the early stages of schistosomiasis, timely administration of PZQ can effectively alleviate the progression of liver fibrosis and, in some cases, lead to near-complete resolution of granulomas. At this stage, egg calcification and replacement of granulomas by thin fibrous scars may be observed. However, delayed PZQ treatment reduces the potential for significant fibrosis regression and increases the risk of developing advanced schistosomiasis [92, 93]. Thus, early antiparasitic chemotherapy, along with strengthened monitoring and assessment of collagen subtype composition, plays a crucial role in halting or even reversing the progression of hepatic fibrosis and improving prognosis in patients.

An increasing number of studies have revealed that the prevalence of schistosomiasis does not always correlate with the severity of hepatic fibrosis in affected populations [94–98]. For example, in Egypt and Sudan, although both the prevalence and intensity of *Schistosoma* infections are lower than those in countries such as Kenya and Mali, the degree of liver fibrosis observed in the population is relatively high [99–103]. Additionally, significant differences in fibrosis progression have been reported even among neighboring regions with similar infection rates [95]. Research by Justin Komguep Nono and colleagues demonstrated that, even within the same endemic area, patients of similar age, sex, socioeconomic status, exposure risk, duration of residence, and PZQ treatment regimens, still show considerable variation in the progression and severity of liver fibrosis [104]. Although differences in infection duration may partially explain these observations [102, 103], accumulating evidence suggests that host genetic background also plays a critical role in the development and progression of hepatic fibrosis [95, 96, 103, 105, 106]. For example, a study conducted in the Poyang Lake region of China revealed that individuals carrying specific alleles of human leukocyte antigen (HLA), such as HLA-DQB1*0303, HLA-DQB1*0609, HLA-DQB1*05031, HLA-DRB1*0901, and HLA-DRB1*1302, were more likely to develop advanced hepatic fibrosis. In contrast, carriers of HLA-DQB1*0601, HLA-DRB1*1501, and HLA-DRB1*1202 presented milder degrees of fibrosis [107, 108]. These findings suggest that host genetic differences may play a regulatory role in the progression of liver fibrosis following the initiation of fibrosis by *Schistosoma* eggs. This raises the question of whether certain individuals possess “schistosomiasis susceptibility genes” that influence fibrotic outcomes independently of ongoing parasitic infection. Candidate genetic factors may include the ABO blood group system or T cell receptor repertoires, which could potentially affect the host’s fibrotic response following antiparasitic therapy.

### Future perspectives and conclusions

PZQ, developed in the 1970s, has been widely used to eliminate intravascular adult schistosomes in infected individuals, saving countless lives worldwide. However, in a subset of patients, hepatic fibrosis continues to progress despite effective antiparasitic chemotherapy, ultimately leading to intractable chronic or advanced schistosomiasis with life-threatening consequences. Thus, parasitological cure alone may no longer be considered the definitive endpoint in schistosomiasis management.

Multiple factors may contribute to the persistent progression of hepatic fibrosis in certain individuals after PZQ treatment. These include differences in the stage of fibrosis at the time of treatment, imbalances in profibrotic and antifibrotic factors in the liver, structural and functional abnormalities in immune organs, co-infection with other pathogens, and variations in host genetic background—all of which may disrupt immune and metabolic homeostasis. Notably, the mechanisms driving fibrosis may vary across individuals. Nevertheless, most current hypotheses regarding these contributing factors are based on epidemiological analyses, clinical observations, and reasoned speculation. Comprehensive and mechanistic studies spanning human populations, animal models, cellular systems, and molecular pathways are still lacking, which significantly hinders the development of effective strategies for managing advanced hepatic schistosomiasis.

Despite these challenges, several potential interventions offer promise. For example, dynamic monitoring of hepatic fibrosis progression in schistosomiasis patients, controlling co-infections with other pathogens, and performing splenectomy in selected cases, may represent viable strategies. The aim of these approaches is to restore hepatic immune and metabolic balance, ultimately halting or reversing fibrotic progression and improving survival in patients with advanced disease. Such efforts are essential to bridging the “last mile” in the post-schistosomiasis control era, offering new hope and direction for both clinical management and public health strategies.

## Data Availability

Not applicable.

## Abbreviations

WHO: World Health Organization
PZQ: Praziquantel
SEA: Soluble egg antigen
HSCs: Hepatic stellate cells
HBV: Hepatitis B virus
HCV: Hepatitis C virus
TIMP-1: Tissue inhibitor of metalloproteinases-1
MMPs: Matrix metalloproteinases
MMP9: Matrix metalloproteinase 9
LPS: Lipopolysaccharide
TLR4: Toll-like receptor 4
MyD88: Myeloid differentiation primary response 88
NF-κB: Nuclear factor kappa-B
TGF-β: Transforming growth factor beta
Smad3: SMAD family member 3
TCRs: Cell receptors
Th2: Helper type 2
RIPK3: Receptor-interacting protein kinase 3
MLKL: Mixed lineage kinase domain-like protein
DAMPs: Damage-ssociated molecular patterns
ROS: Reactive oxygen species
α-SMA: Alpha-Smooth muscle actin
Smad2: SMAD family member 2
Smad4: SMAD family member 4
MAPK: Mitogen-activated protein kinase
ERK: Extracellular signal-regulated kinase
p38: Mitogen-activated protein kinase
JNK: C-Jun N-terminal kinase
CTGF: Connective tissue growth factor
COL1A1: Collagen type I alpha 1 chain
COL1A2: Collagen type I alpha 2 chain
NLRP3: NOD-like receptor family pyrin domain containing 3
HMGB1: High mobility group box 1
RAGE: Receptor for advanced glycation end products
PI3K: Phosphoinositide 3-kinase
AKT: AKT serine/threonine kinase
IL-13: Interleukin 13
IL-4: Interleukin 4
IL-5: Interleukin 5
HLA: Human leukocyte antigen
